# Supplementary material for: Stochastic treatment regimes in climate-health research: Reassessing malaria risk under warming scenarios in Colombia
Source: PLOS Glob Public Health. 2025 Sep 25;5(9):e0005252. doi: 10.1371/journal.pgph.0005252 (PMC12463259; doi:10.1371/journal.pgph.0005252)
Supplement: S1 Text — (DOCX) [file pgph.0005252.s001.docx]

**Temperature regimes analysis**

We demarcated a structural causal model (SCM) to define how the causal mechanism changes under hypothetical interventions on the temperature:

$$W=fW\left( UW \right)$$

$$T=fT\left( W,UT \right)$$

$E=fE\left( T,W,UE \right)$ (1)

Where, $W$ are the confounders, $T$ is the temperature, and $E$ the excess malaria cases. The SCM describes the temporal ordering between the variables, specifies deterministic functions $\left\{ fE, fT, fW \right\}$, generating the variables of the model based on those preceding it and unobserved variables $\left\{ UE, UT, UW \right\}.$

Causal effects are defined in terms of contrasts of hypothetical interventions on the temperature of the SCM (1). The causal effect of different temperature regimes on excess malaria cases can be estimated under the next definitions:

1. Piecewise smooth invertibility: With $\delta$ being a conditional density, for each $w\in W$, assume that the interval $I(w)=(l(w,),u(w))$ may be partitioned into subintervals $I_{\delta,j}(w):j=1,\ldots,J(w)$ such that $d(t,w;\delta)$ is equal to some $d_{j}(t,w;\delta)$ in $I_{\delta,j}(w)$ and $d_{j}(.,w;\delta)$ has an inverse function $b_{j}(\cdot,w;\delta)$ with derivate $b_{j}^{'}(\cdot,w;\delta)$.
2. Randomization assumption: $T_{i}\perp E_{i}^{T_{\delta,i}}\mid W_{i}$ for $i=1,\ldots,n$.
3. **Treatment positivity:** $t_{i}\in\mathcal{T}\Longrightarrow d(t_{i},w_{i})\in\mathcal{T}$ for all $w\in\mathcal{W}$, where $\mathcal{T}$ denotes the support of $T\mid W=w_{i} \forall_{i}=1,\ldots n$.

An efficient influence function was used to develop efficient estimators (i.e., a function that provides the minimum variance, maximizing the accuracy and reliability of statistical inferences) in the non-parametric model $\mathcal{M}$ [41]. In our case we implemented a targeted maximum likelihood (TML) estimator. The TML estimator allows an efficient estimation and inference on the target quantity of interest, which in our analysis was the effect of different temperature regimes on excess malaria cases.

The EIF with respect to the non-parametric model $\mathcal{M}$, may be reduced to the auxiliary covariate [40]:

$H\left( t,w \right)= \frac{go(t- \delta\mid w)}{go(t \mid w)}+1$ (2)

Where $go$ is the exposure model for the observation, and when the treatment $T$lies within the limits defined by the covariate strata $W$ that is, for $T_{i}\in(u(w)-\delta,u(w))$.

The targeted maximum likelihood (TML) estimator can be obtained following the next steps:

1. To build initial estimators $gn$ of $go(T,W)$ and $\bar{Q_{n}}$ of $\bar{Q_{n}} (T,W)$ (the outcome regression function), whit machine learning regression techniques.
2. For each observation $i$, estimate $H_{n}\left( t_{i},w_{i} \right)$ of the auxiliar covariate $H\left( t_{i},w_{i} \right)$.
3. To build a regression model:

$\bar{Q_{\xi,n}}(t,w)=\bar{Q_{n}}(t,w)+\xi H_{n}\left( t,w \right)$ (3)

incorporating $H_{n}$as weights, and estimate the regression model’s parameter $\xi$, obtaining $\xi_{n}$. The outcome of this regression model produces $\bar{Q_{n}^{*}}$.

1. To estimate the TML estimator $\Psi_{n}$ of the effect of temperature on excess cases, defining update $\bar{Q_{n}^{*}}$ of the first estimate $\bar{Q_{n,\xi_{n}}}$ :

$\psi_{n}=\frac{1}{n}\sum_{i=1}^{n} \bar{Q_{n}^{*}}(d(T_{i},W_{i}),W_{i})$ (4)

A more detailed explanation about stochastic treatment regimes can be found in [39].

We estimate the effect on excess malaria cases for the next temperature regimes: 1) Current temperatures (observed temperatures + 0.00001 °C), 2) Observed temperatures + 0.5 °C, 3) Observed temperatures + 1.0 °C, 4) Observed temperatures + 1.5 °C, and 5) Observed temperatures + 2.0 °C. To avoid a failure of memory, we binarized the continuous co-variates. To estimate the effect of the different temperature regimes, we used the R packages tmle3shift version 0.2.1, and sl3 version 1.4.3.
