# Supplementary material for: Stochastic treatment regimes in climate-health research: Reassessing malaria risk under warming scenarios in Colombia
Source: PLOS Glob Public Health. 2025 Sep 25;5(9):e0005252. doi: 10.1371/journal.pgph.0005252 (PMC12463259; doi:10.1371/journal.pgph.0005252)
Supplement: Attachment — Submitted filename: Response to Reviewers.docx [file pgph.0005252.s003.docx]

The authors of this manuscript are grateful for your time in reviewing it. Below, you will find the corresponding corrections and changes, primarily in the Methods and Discussion sections, along with detailed responses and the resubmitted files. Please note that we identified an issue with Figure 3 in the previous version, as it displayed the expected values rather than the Average Treatment Effect. For this reason, we have modified the figure.

1. Does this manuscript meet PLOS Global Public Health’s publication criteria? Is the manuscript technically sound, and do the data support the conclusions? The manuscript must describe methodologically and ethically rigorous research with conclusions that are appropriately drawn based on the data presented.

Reviewer #1: Yes

2. Has the statistical analysis been performed appropriately and rigorously?

Reviewer #1: I don't know

3. Have the authors made all data underlying the findings in their manuscript fully available (please refer to the Data Availability Statement at the start of the manuscript PDF file)?

The PLOS Data policy requires authors to make all data underlying the findings described in their manuscript fully available without restriction, with rare exception. The data should be provided as part of the manuscript or its supporting information, or deposited to a public repository. For example, in addition to summary statistics, the data points behind means, medians and variance measures should be available. If there are restrictions on publicly sharing data—e.g. participant privacy or use of data from a third party—those must be specified.

Reviewer #1: Yes

4. Is the manuscript presented in an intelligible fashion and written in standard English?

PLOS Global Public Health does not copyedit accepted manuscripts, so the language in submitted articles must be clear, correct, and unambiguous. Any typographical or grammatical errors should be corrected at revision, so please note any specific errors here.

Reviewer #1: Yes

5. Review Comments to the Author

Please use the space provided to explain your answers to the questions above. You may also include additional comments for the author, including concerns about dual publication, research ethics, or publication ethics. (Please upload your review as an attachment if it exceeds 20,000 characters)

Reviewer #1: I see this paper more of a confirmation of existing studies on the effects of temperature on causing malaria. In addition to the temperature as the input variables, the approaches consider 11 other atmospheric and oceanic indices acting as confounding factors.

- The study does not address how its aggregate findings might limit conclusions about individual-level malaria risk, which is a fundamental limitation of the ecological study design in terms of its scientific approach and the scope of its inferences. An association found between temperature and malaria incidence at the municipality level might not necessarily hold true at the individual/household level, or that the observed patterns for municipalities do not directly translate to individual risk factors. The study does not address how its aggregate findings might limit conclusions about individual-level malaria risk.

R: We accept the comment and have added a new paragraph in lines 355 - 370 to address this limitation of our research.

- There are other studies considering socio-economic factors and while the authors refer to some effectors of colonization, they were not considered as a variable.

R: We appreciate your comment. For this reason, we have added a new paragraph in lines 402 - 415 to highlight the role of socio-economic factors, such as poverty, in modulating the temperature-malaria relationships.

- The authors do not discuss the potential for selection bias inherent in studying only a subset of municipalities based on their outcome (high malaria incidence). The causal relationship between temperature and malaria transmission, including the optimal temperature range and the Average Treatment Effect (ATE), might differ in municipalities with lower malaria incidence or different epidemiological profiles. Therefore, the findings regarding temperature effects and future warming scenarios, while valuable for the studied areas, may not be broadly generalizable or valid for areas with different baseline malaria burdens or environmental characteristics within Colombia or elsewhere. This constitutes a limitation in the external validation and broader applicability of the scientific findings.

R: We accepted the comment and added a new paragraph in the Discussion section (lines 388 - 401) regarding the implications of the potential selection bias in our research.

- The methodology used (e.g., DAG or causal machine learning) has not been cited and I believe any references to previous work that inspires this work will be appreciated.

R: We are grateful for the comment and have included citations for the methods, such as DAG, causal machine learning, TMLE, STR, and Super Learner, in the Introduction and Methods sections.

6. PLOS authors have the option to publish the peer review history of their article (what does this mean?). If published, this will include your full peer review and any attached files.

Do you want your identity to be public for this peer review? If you choose “no”, your identity will remain anonymous but your review may still be made public.

For information about this choice, including consent withdrawal, please see our Privacy Policy.

Reviewer #1: Yes: Dr. Hamid Mukhtar
