# Supplementary material for: Stochastic treatment regimes in climate-health research: Reassessing malaria risk under warming scenarios in Colombia
Source: PLOS Glob Public Health. 2025 Sep 25;5(9):e0005252. doi: 10.1371/journal.pgph.0005252 (PMC12463259; doi:10.1371/journal.pgph.0005252)
Supplement: Attachment — Submitted filename: Response_to_Reviewers.docx [file pgph.0005252.s004.docx]

Specifically, further explanation on the model and variables are required. In addition, since there are many analysis steps and methods involved, it would be good to provide an overview diagram for the readers to understand how these fit together.

- Line 17, Abstract: Please spell out "approximately" in full.
- R: We accepted the comment, and we have changed approx. to approximately in the Abstract.
- Line 91: It would be good to add a flowchart of the analysis steps and methods used at the start of the Methods section, highlighting the purpose of each step.
- R: We accepted your comment. We have included Figure 1, which contains a flowchart of the analysis steps and methods used.
- Line 141: Please provide details on the 19 bioclimatic variables and the final model formula for the vectors' co-occurrence model. How was variable selection conducted?
- R: We addressed the comment. We modified Section 2.3, Municipality Area with Malaria Vectors’ Co-occurrence (lines 140–189) to include details on bioclimatic variables, the model of vectors' potential distribution, and variable selection.
- Line 186-187: Why did you choose to consider the percentage of area within each municipality suitable for supporting four or more vector species? E.g. why not two or more?
- R: This is an interesting point, and actually, it is possible to consider other percentages of the municipality’s area that are suitable for supporting a different number of vectors, e.g., two or more. However, note that according to the Directed Acyclic Graph (DAG), the potential vector co-occurrence of any number of vector species is a collider in the DAG. This implies that this variable must be excluded from the model to estimate the effect of temperature on malaria cases, in order to avoid inducing collider bias, as mentioned in lines 306-314.
- Line 191-195: Please provide references to support the statement that successive governments have systematically overlooked in terms of social advancement and provision of essential services for communities inhabited mainly by Indigenous and Afro-descendant populations, as a result of Spanish colonizers identifying these areas as having unhealthy climates.
- R: We accepted the comment. We have included two references to support this statement (lines 220-227).
- Line 259: In addition to the code for the causal machine learning analysis, it would be good to provide the code for the vectors' co-occurrence model.
- R: We appreciate the comment. We have added the code for the vector co-occurrence model on GitHub (line 290).
